# Micafungin versus anidulafungin in critically ill patients with invasive candidiasis: a retrospective study

**DOI:** 10.1186/s12879-016-1825-3

**Published:** 2016-09-15

**Authors:** Patrick J. van der Geest, Nicole G. M. Hunfeld, Sophie E. Ladage, A. B. Johan Groeneveld

**Affiliations:** 1 Department of Intensive Care Medicine, Erasmus University Medical Center, ‘s Gravendijkwal 230, 3015 CE Rotterdam, The Netherlands; 2 Department of Pharmacy, Erasmus University Medical Center, ‘s Gravendijkwal 230, Rotterdam, 3015 CE The Netherlands

**Keywords:** Critically ill, Micafungin, Anidulafungin, Invasive candidiasis

## Abstract

**Background:**

In critically ill patients the incidence of invasive fungal infections caused by *Candida spp.* has increased remarkably. Echinocandins are recommended as initial treatment for invasive fungal infections. The safety and efficacy of micafungin compared to caspofungin is similar, but no comparison is made between anidulafungin and micafungin concerning safety and efficacy. We therefore performed a retrospective study to assess these aspects in critically ill patients with invasive candidiasis.

**Methods:**

All patients in the intensive care unit (ICU) with invasive candidiasis, who were only treated with anidulafungin or micafungin, between January 2012 and December 2014 were retrospectively included. Baseline demographic characteristics, infection characteristics and patient courses were assessed.

**Results:**

A total of 63 patients received either anidulafungin (*n* = 30) or micafungin (*n* = 33) at the discretion of the attending intensivist. Baseline characteristics were comparable between the two groups, suggesting similar risk for developing invasive candidiasis. Patients with invasive candidiasis and liver failure were more often treated with anidulafungin than micafungin. Response rates were similar for both groups. No difference was observed in 28-day mortality, but 90-day mortality was higher in patients on anidulafungin. Multivariable cox regression analysis showed that age and serum bilirubin were the best parameters for the prediction of 90-day mortality, whereas APACHE II, Candida score and antifungal therapy did not contribute (*P* > 0.05). None of the patients developed impaired liver function related to antifungal use and no differences were seen in prothrombin time, serum transaminases and bilirubin levels between the groups, after exclusion of patients with liver injury or failure.

**Conclusion:**

Micafungin can be safely and effectively used in critically ill patients with invasive candidiasis. The observed increased 90-day mortality with anidulafungin can be explained by intensivists unnecessarily avoiding micafungin in patients with liver injury and failure.

## Background

The incidence of invasive fungal infections caused by *Candida spp.* is increasing in critically ill patients [1, 2]. The latter is associated with prolonged duration of hospitalization and higher mortality rates [3–5]. The mortality of invasive candidiasis is directly correlated with a delay in starting antifungal therapy and therefore early treatment with an appropriate antifungal drug is mandatory [6, 7].

A relatively novel class of antifungal agents are echinocandins which have activity against a broad spectrum of *Candida spp.,* including *C. glabrata* and *C. krusei*, against which fluconazole has less activity [8]. Echinocandins are recommended as initial treatment for invasive candidiasis in patients with moderate to severe illness, keeping fluconazole reserved for less critically ill patients [9]. The recommendation are based on prospective randomized clinical trials which demonstrated that all three echinocandins are at least as effective as fluconazole for the treatment of invasive candidiasis [10–15]. The antifungal activity, pharmacokinetics and toxicity profile of each echinocandin is slightly different, but the relevance of this finding remains unclear [16]. Both caspofungin and micafungin undergo hepatic metabolism, in contrast to anidulafungin, which undergoes spontaneous degradation [17]. Concerns about possible hepatotoxicity of micafungin have been raised which may affect its use in daily practice [2]. Two randomized controlled trials [16, 18] and one retrospective observational trial [19], compared the safety and efficacy of micafungin versus caspofungin in the treatment of invasive candidiasis, showing similar results in the safety and efficacy of micafungin compared with caspofungin. One systematic review including 8,000 patients [20] and one observational cohort study with 8,696 patients [21], evaluated the safety of micafungin versus other echinocandins, showing no increased risk of hepatic injury by micafungin. Both studies only evaluated the safety concerning hepatotoxicity of antifungal medication in mostly non-ICU patients, but did not evaluate mortality or efficacy.

As far as we are aware there are no studies comparing the efficacy and safety of micafungin versus anidulafungin in critically ill patients with invasive candidiasis. We performed a retrospective study to compare the safety and efficacy of micafungin versus anidulafungin in critically ill patients with invasive candidiasis.

## Methods

### Patients

We retrospectively gathered data from the patients’ medical records using a predefined checklist. Between January 1, 2012, and January 1, 2015, all patients over the age of 18 with invasive candidiasis and who only received anidulafungin or micafungin as systemic antifungal treatment in the Intensive Care Unit (ICU) of the Erasmus University Medical Center Rotterdam, were considered for participation. Candidemia was defined as at least one positive blood culture for *Candida spp.* drawn from a peripheral vein. Invasive candidiasis was defined as a positive culture with *Candida spp.* obtained from a normally sterile site, such as pleural or peritoneal fluid, in the context of pleural exsudate/empyema, and secondary or tertiary peritonitis following a ruptured viscus and surgery, respectively [22]. In addition, patients needed to have one or more of the following signs and symptoms of infection: fever or hypothermia; hypotension; localized signs and symptoms of inflammation; or radiological findings of invasive candidiasis. Between January 2012 and December 2014 there were 124 patients diagnosed with invasive candidiasis of whom 20 received caspofungin, 38 stepped-down to fluconazole and 3 received both antifungals. In total there were 63 patients with invasive *Candida spp.* infection who only received anidulafungin or micafungin. The Dutch law states that informed consent is not required in case of retrospective analysis in which data collected during routine clinical care were used and anonymously analyzed.

### Clinical protocol

Patients were taken care of by attending intensivists according to national and local guidelines. In our center selective decontamination of the digestive tract (SDD) is routinely used for patients with an expected duration of mechanical ventilation for more than 48 h. This involves administration of an oral paste and of a suspension via the nasogastric tube, containing the non-absorbable antibiotics tobramycin, amphotericin-B and colistin. Patients also received cefotaxime intravenously at 4 times 1 g a day for a three-day period. Inventory cultures are taken of the throat, tracheal aspirates, and rectum as part of this protocol on admission. To monitor the effect of SDD treatment, surveillance cultures (from throat, tracheal aspirates, and rectum) were routinely performed three times per week. All SDD cultures were screened for the presence of Gram-negative rods, and yeast that were identified to the species level (see below). In case a patient is suspected for having an infection, additional cultures (besides the routinely SDD cultures) can be taken from the possible source of infection, which includes the use of blood cultures. All materials obtained from a normally sterile site were cultured onto relevant agar plates for the detection of both bacteria and yeast. Blood culture bottles, specific for the recovery of yeast (BACTEC Mycosis IC/F), that became positive were subcultured onto chocolate agar, Sabouraud agar, and CHROMagar^TM^ to ensure purity or mixed infection and differentiation of yeast, and incubated at 35 °C. Auxacolor (Sanofi Diagnostics Pasteur) or MALDI-TOF was used to identify the species level of a *Candida* colony as soon as visible growth from a normally sterile site was observed. Susceptibility testing of isolates obtained from normally sterile sites was performed using a CLSI broth microdilution method (Sensititre®, Thermoscientific, USA) and results for azoles, amphotericin B and caspofungin, were reported according to revised species-specific CLSI clinical breakpoints. For fluconazole, *C. albicans* was considered susceptible if the minimum inhibitory concentration (MIC) was ≤2 mg/L, and reduced susceptibility was defined as a MIC of ≥4 mg/L [23], after 24–48 h of growth. The decision to start an echinocandin was taken by the attending intensivist in collaboration with infectious disease specialist. The initiation of antifungal therapy was based on clinical signs (i.e. fever, hypothermia, hypotension, leukocytosis or leukopenia), risk factors for invasive candidiasis, culture results, radiological findings of invasive candidiasis, and according to published criteria [9]. Caspofungin was introduced in 2001, followed by anidulafungin and micafungin respectively. In case of severe liver injury and liver failure, which was defined as the presence of clinical signs and symptoms of an abnormal liver function (increased liver enzymes, hyperbilirubinemia, coagulopathy and encephalopathy), intensivists prefer anidulafungin over micafungin. In all other instances, the choice between anidulafungin or micafungin was at the discretion of the attending intensivist. For anidulafungin patients received an intravenous daily dose of 100 mg after an initial single loading dose of 200 mg. Micafungin was administered at a dose of 100 mg intravenous once daily without a loading dose. No dose adjustment was needed for body weight or impaired renal or hepatic function. Drainage of suspected pus collections as well as removal of IV catheters suspected to be the origin of infection was routinely done. The duration of the antifungal therapy was decided in close collaboration with the infectious diseases physician, based on Dutch invasive fungal infection guidelines which take several factors into account, such as duration of positive cultures, the certainty of good drainage and clinical improvement (absence of fever for >24 h, haemodynamic stability, and neutropenia) [9, 24, 25].

### Study protocol and data collection

Demographic data and clinical date were recorded on admission, including severity of illness scores, risk factors for invasive *Candida spp.* infection (neutropenia, recent surgery, diabetes mellitus, cancer, mechanical ventilation, renal replacement therapy, total parenteral nutrition, transplant and central venous catheters), the duration of ICU stay, and mortality at day 28 and 90 after start of antifungal therapy. Patients were checked for abnormal liver function during antifungal therapy, serum aspartate transaminase (AST) and serum alanine transferase (ALT) were recorded at start and stop of echinocandin treatment. Serum bilirubin, prothrombin time (PT), AST, and ALT peak values were recorded daily during echinocandin treatment. At the start of echinocandin treatment a *Candida* score was calculated for each patient to help, when >3, establishing the risk for invasive candidiasis vs colonization [26]. The primary site of infection and the obtained culture results were recorded. A global response at the end of treatment was defined as both clinical success (cure – resolution of signs and symptoms of *Candida spp.* infection, or improvement – incomplete resolution of signs and symptoms of *Candida spp.* infection) and microbiological success (2 negative follow-up cultures for *Candida spp.*, or presumed eradication when the follow-up culture was not available and clinical response was defined as cure or improvement at the end of treatment). The duration of *Candida spp.* infection was defined as the period between the first positive culture and the first negative culture or ICU death or discharge. Duration of treatment was defined as the period between initiation and stop of antifungal therapy or ICU death or discharge.

### Statistical analysis

Continuous variables were presented with median and interquartile range because of the not normal distribution (Kolmogorov-Smirnov test, *P* < 0.05). Continuous data were examined with The Mann–Whitney U test and categorical data with the Fisher exact test. The Kaplan-Meier estimation of survival curves (compared by log rank tests) and multivariable cox regression were used for survival analysis. All reported *P* values are two-tailored. Statistical significance was set at *P* < 0.05.

## Results

Sixty-three patients with invasive candidiasis received either anidulafungin (*n* = 30) or micafungin (*n* = 33). Patients using anidulafungin were more often on renal replacement therapy (Table 1).

**Table 1:** Baseline demographic and clinical characteristics

	Anidulafungin	Micafungin	*P*
	(*n* = 30)	(*n* = 33)	
On admission			
Age (years)	59 (20)	62 (20)	0.36
Gender (male)^a^	19 (63)	26 (79)	0.18
APACHE II score	26 (10)	23 (8)	0.13
SOFA score	10 (9)	9 (3)	0.63
Reasons of ICU admission^a^			0.14
Suspected sepsis	9 (30)	11 (33)	
Respiratory failure	2 (7)	6 (19)	
Renal failure	1 (3)	0 (0)	
Liver failure	4 (13)	0 (0)	
CPR	2 (7)	2 (6)	
Shock	5 (17)	3 (10)	
Postoperative	7 (23)	11 (34)	
At start of echinocandin			
Risk factors for invasive candidiasis^a^			
Neutropenia	3 (10)	2 (6)	0.57
Broad spectrum antibiotics	18 (60)	15 (45)	0.25
Immunosuppression	4 (13)	3 (9)	0.60
Steroids	16 (53)	12 (36)	0.18
TPN	4 (13)	11 (33)	0.07
Recent Surgery	8 (27)	15 (45)	0.13
DM II	11 (37)	7 (21)	0.18
Malignancy	5 (17)	11 (33)	0.13
Transplant	5 (17)	4 (12)	0.61
CVVH	19 (63)	7 (21)	0.001
CVC	29 (97)	29 (88)	0.20
Mechanical ventilation	27 (90)	32 (97)	0.26
Duration between admission and start echinocandin (days)	3 (4)	2 (2)	0.17

Numbers (percentage) ^a^or median (interquartile range), where appropriate

List of abbreviations: *APACHE II* Acute Physiology and Chronic Health Evaluation II, *CPR* cardiac pulmonary resuscitation, *CVVH* continuous venovenous haemofiltration, *CVC* central venous catheter, *DM II* diabetes mellitus type II, *ICU* intensive care unit, *TPN* total parenteral nutrition

### Infection and treatment characteristics

**Fig. 1:**
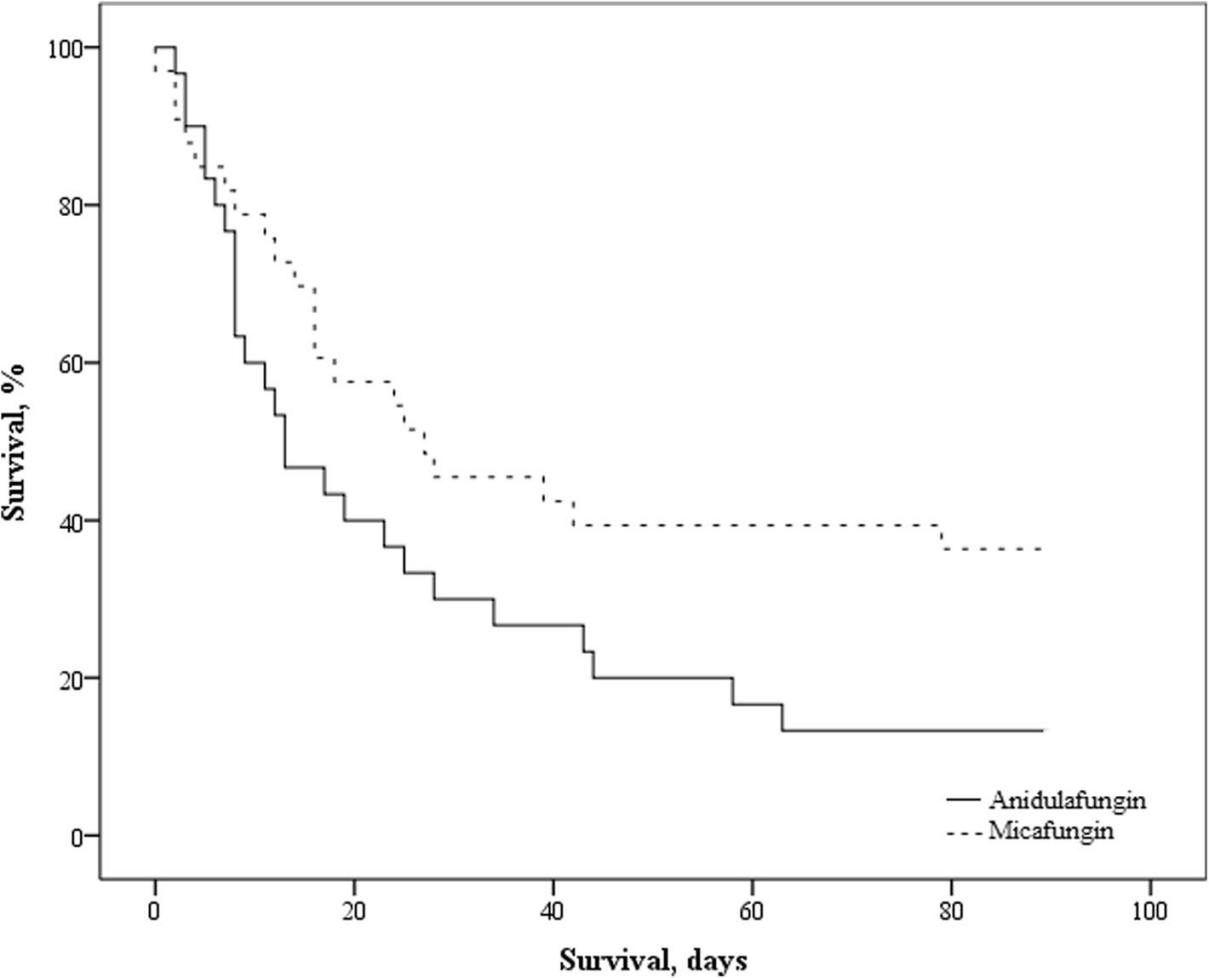
Kaplan-Meier survival curve up to day 90 after initiation of an echinocandin, *P* = 0.04 (log rank test). Numbers at risk, micafungin group: 33, 19, 14, 13, 12. Numbers at risk, anidulafungin group: 30, 12, 8, 5, 4

Of the 63 patients, 22 patients had candidemia, 37 patients had abdominal and 4 patients had pleural infection (Table 2). The average *Candida* score was 3 for both groups. Anidulafungin was initiated at median day 3 and micafungin was initiated at median day 2 after ICU admission. There were no echinocandin-resistant strains. Four *C. albicans* isolates were considered less sensible to fluconazole with an average clinical breakpoint of 100 mg/L. There was no difference in the duration of treatment and infection, and response rates, even when corrected for liver failure. Both groups had a similar duration of ICU stay. No difference was observed in 28-day mortality, but 90-day mortality was higher in patients on anidulafungin (Table 2 and Fig. 1). Multivariable Cox regression analysis showed that age (hazard ratio 1.13, 95 % confidence interval 1.02–1.23, *P* = 0.02) and serum bilirubin (hazard ratio 1.06, 95 % confidence interval 1.01–1.12, *P* = 0.03) were the best parameters for the prediction of 90-day mortality, whereas APACHE II, *Candida* score and antifungal therapy did not contribute (*P* > 0.05).

**Table 2:** Infection and treatment characteristics

	Anidulafungin	Micafungin	*P*
	(*n* = 30)	(*n* = 33)	
*Candida* score at start	3 (2)	3 (3)	0.71
Source of isolates^a^			0.25
Blood	10 (33)	12 (36)	
Intra-abdominal fluid	17 (57)	20 (63)	
Pleural fluid	3 (10)	1 (3)	
*Candida spp.* ^*a*^			0.46
*Candida albicans*	8 (27)	3 (9)	
*Candida dublienis*	0 (0)	2 (6)	
*Candida glabrata*	17 (57)	21 (66)	
*Candida krusei*	3 (10)	3 (10)	
*Candida parapsilosis*	1 (3)	2 (6)	
*Candida tropicalis*	1 (3)	2 (6)	
Global response^a^	21 (67)	23 (70)	0.80
Clinical response^a^	24 (80)	28 (85)	0.62
Microbial response^a^	21 (70)	24 (73)	0.81
Gaining negative cultures^a^	18 (60)	20 (60)	0.96
Duration of infection (days)	3 (5)	3 (5)	0.80
Duration of treatment (days)	12 (8)	14 (9)	0.40
Length of ICU stay (days)	13 (15)	14 (21)	0.64
Mortality day 28 after start^a^	20 (67)	18 (55)	0.33
Mortality day 90 after start^a^	26 (87)	21 (64)	0.04

Numbers (percentage) ^a^or median (interquartile range), where appropriate

Abbreviations: *ICU* intensive care unit

### Liver enzymes and function

Patients with invasive candidiasis and liver failure were more often treated with anidulafungin than micafungin. None of the patients in this study developed liver failure or elevated liver enzymes requiring cessation of treatment related to the use of an echinocandin. PT, AST, ALT and bilirubin levels tended to be higher in patients treated with anidulafungin. When excluding liver failure, no differences were seen in PT, AST, ALT and bilirubin levels between the groups (Table 3).

**Table 3:** Liver enzymes and function

A	Anidulafungin	Micafungin	*P*
	(*n* = 30)	(*n* = 33)	
Liver failure^a^	9 (30)	2 (6)	0.01
Increased serum AST (U/L) after start^a^	13 (43)	12 (36)	0.58
Increased serum ALT (U/L) after start^a^	13 (43)	10 (30)	0.29
Serum AST (U/L) at start	95 (282)	55 (76)	0.09
Serum ALT (U/L) at start	56 (125)	39 (59)	0.13
Serum AST (U/L) at stop	115 (1011)	52 (108)	0.17
Serum ALT (U/L) at stop	75 (312)	45 (68)	0.14
Serum AST (U/L) peak	285 (228)	116 (181)	0.11
Serum ALT (U/L) peak	150 (404)	70 (126)	0.15
Serum bilirubin (μmol/L) peak	71 (192)	15 (29)	0.05
Prothrombin Time (sec) peak	18.3 (15.4)	17.8 (8.0)	0.27
B	Anidulafungin	Micafungin	*P*
	(*n* = 21)	(*n* = 31)	
Increased serum AST (U/L) after start^a^	8 (38)	11 (35)	0.85
Increased serum ALT (U/L) after start^a^	7 (33)	9 (29)	0.74
Serum AST (U/L) at start	61 (98)	55 (73)	0.82
Serum ALT (U/L) at start	43 (75)	39 (54)	0.89
Serum AST (U/L) at stop	44 (141)	49 (79)	0.98
Serum ALT (U/L) at stop	41 (83)	45 (54)	0.99
Serum AST (U/L) peak	129 (267)	113 (158)	0.88
Serum ALT (U/L) peak	69 (209)	61 (119)	0.95
Serum bilirubin (μmol/L) peak	20 (105)	14 (27)	0.74
Prothrombin Time (sec) peak	16.6 (7.5)	17.8 (5.3)	0.68

Numbers (percentage) ^a^or median (interquartile range). Liver enzymes and function in all patients who were treated with anidulafungin and micafungin (A) and in patients without liver failure (B)

Abbreviations: *ALT* alanine transaminase, *AST* aspartate transaminase

### Costs

Table 4 describes the involved costs for both treatment strategies. The total treatment costs per patient seemed to be higher for micafungin, because of the longer treatment period. However, the treatment costs per patient per day were lower for micafungin.

**Table 4:** Treatment costs

	Anidulafungin	Micafungin
	(*n* = 30)	(*n* = 33)
Total treatment days	354	429
Total drug dose (mg)	41.600	42.900
Total drug costs (€)	183,805.44	209,553.63
Drug costs per patient (€)	6,126.84	6,350.11
Drug costs p.p.p.d. (€)	519.22	488.47

Micafungin costs € 460.82 (excluding VAT) per 100 mg ampoule, € 488.47 (including 6 % VAT)

Anidulafungin costs € 416.83 (excluding VAT) per 100 mg ampoule, € 441.84 (including 6 % VAT)

Abbreviations: *p.p.p.d.* per patient per day

## Discussion

This study suggests that in critically ill patients with candidemia or invasive candidiasis, the safety and efficacy of the treatment with micafungin was similar to that of anidulafungin. No differences were seen in response rates, liver function and enzymes, and mortality. Treatment with micafungin seems to be less expensive then that with anidulafungin.

Echinocandins inhibit synthesis of the β-(1–3)-D-glucan compound of the fungal cell wall and are considered as safe drugs [2, 17]. The overall efficacy between the three echinocandins is comparable, showing only little difference [27]. Nevertheless, the EMA still recommend to only use micafungin in case other antifungals are not appropriate, as rat experiments suggested a potential risk for the development of liver tumors [27]. However, these results were obtained from studies using high dosages for prolonged time in male animals, similar effects were not reported by other studies on humans or animals [17]. Both caspofungin and micafungin undergo hepatic metabolization, in contrast to anidulafungin, which undergoes spontaneous degradation [17]. Transient elevation of liver enzymes occurs in 2 to 15 % of patients treated with an echinocandin [28]. In this study we found an elevation in serum AST of 38 % and 35 %, and an elevation in serum ALT of 33 % and 29 %, in patients treated with anidulafungin or micafungin, respectively. The observed incidence of elevated liver enzymes in this study is much higher, but equal between both groups of echinocandins, but we only looked at elevated liver enzymes in general and not specifically caused by the echinocandins. Abnormal liver function tests can be found in up to 61 % of critically ill patients, as caused by sepsis, drugs or ischemia [29]. Our results suggest that micafungin is as safe as anidulafungin concerning hepatotoxicity. The results are in line with two previous studies, which both concluded that anidulafungin and micafungin had a low risk of elevated liver enzyme levels not requiring the cessation of treatment [20, 21]. Both studies only evaluated the safety concerning hepatotoxicity of antifungal medication in mostly non-ICU patients, but did not evaluate mortality or efficacy, as we did. Therefore, this study adds important information about the safety and efficacy of micafungin compared with anidulafungin in critically ill patients. At day 28 the overall mortality was 60 % which is comparable to the mortality described in other studies [30, 31]. The data suggest that higher 90-day mortality with anidulafungin than micafungin reflects more severe underlying liver disease rather than effect of treatment itself.

The type and duration of treatment of invasive candidiasis depends on culture results and sensitivity testing, the extent of organ involvement and patients’ clinical condition [9, 24]. In patients with invasive fungal infections the recommended length of therapy is 14 days after the first negative blood culture [9, 24], which is in line with the observed median length of treatment of 12–14 days in this study. The overall response rates in this study are comparable to those found in other performed studies [11, 32]. The overall response rate of micafungin is comparable to that of anidulafungin. Our retrospective data, concerning the safety and efficacy of micafungin are in line with a recent performed study, which evaluated the safety and efficacy of micafungin monotherapy in critically ill patients with cancer and invasive candidiasis [32]. However, the study did not make a comparison with anidulafungin, as we did. The pharmacokinetics of micafungin are very well defined in non-critically ill patients and seems to be similar in critically ill patients [33]. In critically ill patients micafungin reaches a steady state by day 3, without the need for a loading dose, in contrast to caspofungin and anidulafungin [9, 24, 25, 33]. Dose adaptations are not required for body weight and in patients with renal or hepatic impairment and renal replacement therapy [33]. The involved costs per patient per day of treatment with micafungin seems to be a bit lower compared with anidulafungin.

One of the limitations of this study is the relatively small number of patients included, so that results should be regarded as preliminary. This study enrolled 63 patients with invasive candidiasis caused by *Candida spp.* in a four year period in a tertiary care ICU with 2,000 admissions per year. Hence, the reported incidence of invasive candidiasis in a large review was 5 to 10 cases per 1,000 ICU admission, which is comparable with our reported incidence of 6 per 1,000 ICU admissions [34]. Second, because of the retrospective design we cannot exclude that the use of either agent may have been subject to bias.

## Conclusion

In conclusion, our results suggest that micafungin can be safely and effectively used in critically ill patients with candidemia and invasive candidiasis.

## Abbreviations

ALT: serum alanine transferase
APACHE II: The acute physiology and chronic health evaluation II serum aspartate transaminase
AST: Serum aspartate transaminase
EMA: European medicines agency
FDA: Food and drug administration
ICU: Intensive care unit
IV: Intravenous
MIC: Minimum inhibitory concentration
PPPD: Per patient per day
PT: Prothrombin time
SDD: Selective decontamination of the digestive tract
SOFA: Sequential organ failure assessment
SPP: Species
